# DREISS: Using State-Space Models to Infer the Dynamics of Gene Expression Driven by External and Internal Regulatory Networks

**DOI:** 10.1371/journal.pcbi.1005146

**Published:** 2016-10-19

**Authors:** Daifeng Wang, Fei He, Sergei Maslov, Mark Gerstein

**Affiliations:** 1 Department of Biomedical Informatics, Stony Brook University, Stony Brook, New York, United States of America; 2 Stony Brook Cancer Center, Stony Brook Medicine, Stony Brook, New York, United States of America; 3 Biology Department, Brookhaven National Laboratory, Upton, New York, United States of America; 4 Department of Bioengineering and Carl R. Woese Institute for Genomic Biology, University of Illinois at Urbana-Champaign, Urbana, Illinois, United States of America; 5 Program in Computational Biology and Bioinformatics, Yale University, New Haven, Connecticut, United States of America; 6 Department of Molecular Biophysics and Biochemistry, Yale University, New Haven, Connecticut, United States of America; 7 Department of Computer Science, Yale University, New Haven, Connecticut, United States of America; National Center for Biotechnology Information (NCBI), UNITED STATES

## Abstract

Gene expression is controlled by the combinatorial effects of regulatory factors from different biological subsystems such as general transcription factors (TFs), cellular growth factors and microRNAs. A subsystem’s gene expression may be controlled by its internal regulatory factors, exclusively, or by external subsystems, or by both. It is thus useful to distinguish the degree to which a subsystem is regulated internally or externally–e.g., how non-conserved, species-specific TFs affect the expression of conserved, cross-species genes during evolution. We developed a computational method (DREISS, dreiss.gerteinlab.org) for analyzing the Dynamics of gene expression driven by Regulatory networks, both External and Internal based on State Space models. Given a subsystem, the “state” and “control” in the model refer to its own (internal) and another subsystem’s (external) gene expression levels. The state at a given time is determined by the state and control at a previous time. Because typical time-series data do not have enough samples to fully estimate the model’s parameters, DREISS uses dimensionality reduction, and identifies canonical temporal expression trajectories (e.g., degradation, growth and oscillation) representing the regulatory effects emanating from various subsystems. To demonstrate capabilities of DREISS, we study the regulatory effects of evolutionarily conserved vs. divergent TFs across distant species. In particular, we applied DREISS to the time-series gene expression datasets of *C*. *elegans* and *D*. *melanogaster* during their embryonic development. We analyzed the expression dynamics of the conserved, orthologous genes (orthologs), seeing the degree to which these can be accounted for by orthologous (internal) versus species-specific (external) TFs. We found that between two species, the orthologs have matched, internally driven expression patterns but very different externally driven ones. This is particularly true for genes with evolutionarily ancient functions (e.g. the ribosomal proteins), in contrast to those with more recently evolved functions (e.g., cell-cell communication). This suggests that despite striking morphological differences, some fundamental embryonic-developmental processes are still controlled by ancient regulatory systems.

## Author Summary

The dynamics of a biological system can be controlled by its own internal mechanisms and external perturbations. To gain intuition on this, we may draw a comparison with a mass hanging from a spring. The mass will move naturally by itself but its dynamics is also affected by one’s pulling it. That is, the dynamics of the mass is governed by the effect of the external perturbations superimposed on the internal mechanism of the spring (i.e. Hooke’s law). Similarly, given a group of genes, their temporal gene expression dynamics can be controlled by both transcription factors inside the group and external regulatory factors. Therefore, it is useful to identify the expression dynamics that are exclusively controlled by internal or external factors and compare them across various systems. While state-space models have been widely used to decouple the internal and external effects in physical systems, such as the mass and spring, typical biological systems do not have enough time samples to infer all the model’s parameters, and applications of state-space models were not very effective in these instances. Hence, we developed a general-purpose computational method by integrating state-space models and dimensionality reduction to identify temporal gene expression patterns driven by internal and external regulatory networks. We applied our method to the embryonic developmental datasets in the worm and fly (and also in a human cancer context). We successfully identified the temporal expression dynamics of cross-species conserved genes that were driven by conserved and species-specific regulatory networks.

This is a *PLOS Computational Biology* Methods paper.

## Introduction

Gene regulatory networks systematically control the gene expression dynamics. These networks are highly modular, and consist of various sub-networks. Each sub-network contains a number of regulatory factors representing a subsystem that drives specific gene regulatory functions [1,2]. The subsystems interact with one another, and work together to carry out the entire gene regulatory function. For example, the gene expression in embryogenesis is controlled by the combinatorial effects of various regulatory subsystems composed of complex evolutionary regulatory networks [3]. These regulatory subsystems drive very diverse developmental programs, from the highly conserved (e.g. DNA replication) to the species-specific (e.g. body segmentation). As such the orthologous genes that are evolutionary conserved genes across species can therefore be regulated by both orthologous and species-specific transcription factors (TFs) [4]. The orthologous TFs form an “internal” regulatory network, while the species-specific TFs form an “external” one. Unfortunately, existing experimental gene expression data cannot decouple the expression components that are driven by the different subsystems. Thus, computational methods are required to assess the contribution from each factor or subsystem from the gene expression data. In this study, we propose a novel computational method, DREISS—dynamics of gene expression driven by external and internal regulatory networks based on state space model. Using DREISS, we are able to identify temporal gene expression dynamic patterns for evolutionarily conserved genes during embryonic development, as driven by conserved and species-specific regulatory subsystems. These results advance our current understanding of gene regulatory networks during evolution, as well as the differentiation during development.

Developmental gene regulatory networks control gene expression during the developmental processes. These particular regulatory networks have evolved, making it difficult to understand their regulatory mechanisms at the system level. Hence, one typically compares developmental gene expression across species to infer biological activities of developmental gene regulatory networks. For example, embryogenesis provides a platform to study the evolution of gene expression between different species. Recent work has showed that significant biological insight can be gained by cross-species comparisons of the expression profiles during embryogenesis for worms [5], flies [6], frogs [7] and several other vertebrates [8]. It was found that the orthologous genes have minimal temporal expression divergence during the phylotypic stage, a middle phase during the embryonic development across species within the same phylum. These patterns are often characterized as “hourglass” [9]. In addition, the conserved hourglass patterns were observed even within a single species while comparing the developmental gene expression data across distant species, such as worm and fly [10]; i.e., the expression divergence among evolutionarily conserved genes become minimal during the phylotypic stage in both worm and fly. However, much less is known about how the orthologous genes in each species eventually contribute to their species-specific phenotypes due to the lack of appropriate computational approaches. Thus, we aim to use DREISS to discover the components of the orthologous gene expression during embryonic development driven by species-specific transcription factors.

The state-space model has been widely used in engineering [11], and also in biology for the analysis of gene expression dynamics [12–14]. It models the dynamical system output as a function of both the current internal system state and the external input signal. A well-known example in engineering is the vehicle cruise control system where the system state can be the vehicle’s speed. Based on the road conditions, the cruise control requires various fuel amounts in order to keep the desired speed level. In biology, we can look at the transcription factors and microRNAs as internal and respectively external regulatory factors of the protein-coding genes expression (See more internal-external examples in S1 Table). Similarly, the state-space model can be applied for studying the expression of orthologous genes at different developmental stages using information regarding their expression (internal) and species-specific regulatory factors (external) at the current known developmental stage. Unlike earlier studies that calculate the expression correlation between individual genes, the state-space model predicts the temporal causal relationships at the system level; i.e., the state at a time is determined by the state and external input at the previous time. The earlier work applied the state-space model to study the gene expression dynamics focusing on small-scale systems, and did not explore the analytic dynamic characteristics of the inferred state-space models. The complex and large-scale biological datasets, especially temporal gene expression data, are very noisy, and high dimensional (i.e., the number of genes is much greater than the number of time samples), thereby preventing an accurate estimation of the state-space model’s parameters. The dimensionality reduction techniques have thus been used to project high-dimensional genes to low-dimensional meta-genes (i.e., the selected features representing de-noised and systematic expression patterns [1,15,16]) as well as the principal dynamic patterns for those meta-genes [17,18]. Using DREISS, we are able to apply the dimensionality reduction to the gene expression data, and develop an effective state-space model for their meta-genes, and then identify a group of canonical temporal expression trajectories representing the dynamic patterns driven by the effective conserved and species-specific meta-gene regulatory networks according to the model’s analytic characteristics. These dynamic patterns reveal temporal gene expression components that are controlled by conserved or species-specific GRNs.

DREISS is a general-purpose tool and can be used to study the gene regulatory effects from any different subsystems for a given group of genes. As an illustration, we applied DREISS to the gene expression data during embryonic development for two model organisms, worm (*Caenorhabditis elegans*) and fly (*Drosophila melanogaster*). In both species, we were able to identify the expression patterns of worm-fly orthologs driven by the conserved regulatory network consisting of the worm-fly orthologous TFs (i.e., the conserved regulatory subsystems between two species), as well as the worm/fly-specific regulatory network consisting of non-orthologous TFs (i.e., the species-specific regulatory subsystem). Our results reveal that, in addition to executing conserved developmental functions between worm and fly, their orthologous genes are also regulated by species-specific TFs to involve in species-specific developmental processes. In summary, DRIESS provides a framework to analyze both distantly and closely related species allowing for a better understanding of the gene regulatory mechanisms during development.

## Methods

### DREISS consists of five major steps as detailed in Fig 1

**Step A**: DREISS models temporal gene expression dynamics using state-space models in control theory. In this step, we need to define the internal and external groups of genes and input their time-series gene expression data that we are interested to study. We assume that the time-series gene expression data fits a state-space module. In the state-space model, the “state” refers to the expressions for a large group of genes of interest, such as the worm-fly orthologous genes investigated here. The “control” refers to any other group of genes that contribute to the gene expression of the “state”, such as the species-specific TFs contributed to control orthologous gene expression.
**Step B**: Due to the limited number of temporal samples in gene expression experiments, we do not have enough data to accurately estimate the parameters of the state-space models that capture interactions among hundreds of genes. Therefore, DREISS projects high-dimensional gene expression space to lower-dimensional meta-gene expression spaces using dimensionality reduction techniques.
**Step C**: DREISS derives the effective state-space models for meta-genes so that model parameters can be estimated.
**Step D**: DREISS identifies the meta-gene expression dynamic patterns; i.e., canonical temporal expression trajectories driven by “state” (internal) and by “control” (external) based on the analytic solutions of the estimated models.
**Step E**: Finally, DREISS calculates the gene coefficients over canonical temporal expression trajectories based on linear transformations between genes and meta-genes. DREISS also allows us to compare the dynamic expression patterns of multiple datasets with samples taken at different times. We describe each DREISS step in detail as follows.

**Fig 1:**
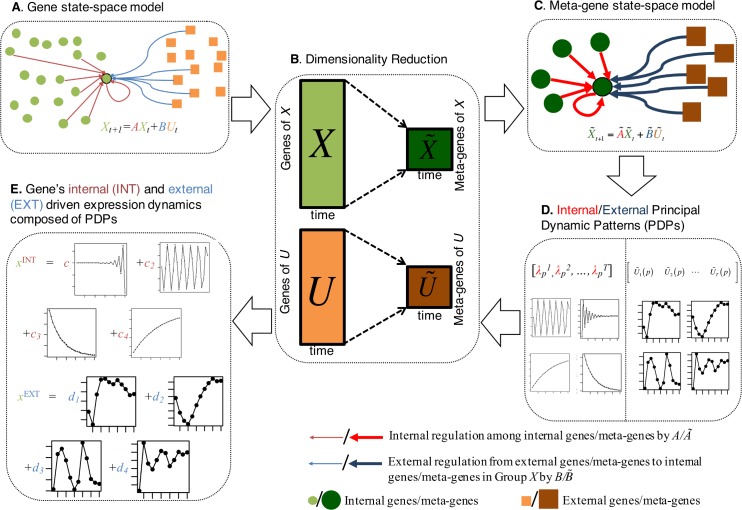
DREISS workflow. **(A)** DREISS models temporal gene expression dynamics using state-space models in control theory. The “state” refers to the expressions for a large group of genes of interest, such as the worm-fly orthologous genes investigated here. The “control” refers to any other group of genes that contribute to gene expressions of the “state”, such as the species-specific TF studied here. **(B)** it then projects high-dimensional gene expression space to lower-dimensional meta-gene expression spaces using dimensionality reduction techniques. **(C)** it derives the effective state-space models for meta-genes so that model parameters can be estimated. **(D)** it then identifies the meta-gene expression dynamic patterns; i.e., canonical temporal expression trajectories driven by “state” (internal) and by “control” (external) based on the analytic solutions to estimated models. **(E)** it finally calculates the coefficients of genes for the dynamic patterns of linear transformations between genes and meta-genes.

### State-space models for temporal gene expression dynamics

**Fig 2:**
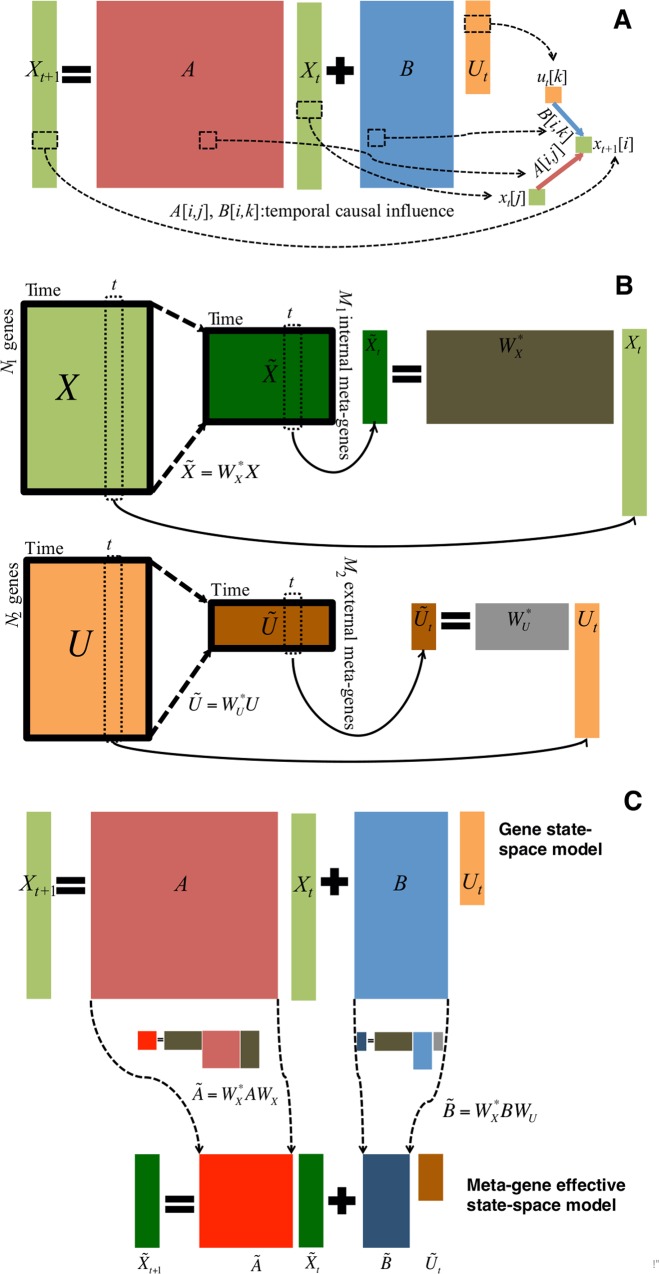
State space model for genes and the effective model for meta-genes. **(A)** linear state space model for a given subsystem’s gene expression; i.e., linear first-order difference equations in Eq 1, is used to formulate temporal gene expression dynamics for a given subsystem, the internal group Ω (comprising *N*_1_ genes) with external regulations from the external group Ψ (comprising *N*_*2*_ genes) at time points 1, 2, …, *T*. The vector $X_{t}\in R^{N_{1}\times 1}$, the “state”, includes *N*_1_ gene expression levels at time *t* in Ω, and the vector $U_{t}\in R^{N_{2}\times 1}$, the “input or control”, includes *N*_2_ gene expression levels at time *t* in Ψ. The system matrix $A\in R^{N_{1}\times N_{1}}$ captures internal causal interactions among genes in Ω (i.e., the *i*^th^, *j*^th^ element of *A*, *A*_*ij*_ describes the contribution from the *j*^th^ gene expression at time *t* to the *i*^th^ gene expression at the next time *t*+1). The control matrix $B\in R^{N_{1}\times N_{2}}$ captures external causal regulations from the genes in Ψ to genes in Ω (i.e., the *i*^th^, *j*^th^ element of *B*, *B*_*ij*_ describes the contribution from the *j*^th^ gene expression in Ψ at time *t* to the *i*^th^ gene expression in Ω at the next time *t*+1). **(B)** Meta-gene expression levels. The meta-gene expression levels are obtained by ${\tilde{X}}_{t}=W_{X}^{*}X_{t};{\tilde{U}}_{t}=W_{U}^{*}U_{t}$, where ${\tilde{X}}_{t}\in R^{M_{1}\times 1}$, the “meta-gene state”, includes *M*_1_ (<< *N*_1_ and <*T*) meta-gene expression levels; i.e., the first *M*_1_ elements of the *t*^th^ row of the matrix whose columns are right-singular vectors of the matrix [*X*_1_ *X*_2_ ⋯ *X*_*T*_] in Ω by the singular value decomposition (SVD) [19]; the vector ${\tilde{U}}_{t}\in R^{M_{2}\times 1}$, the “meta-gene input or control”, includes *M*_2_ (<< *N*_2_ and <*T*) meta-gene expression levels (i.e., the first *M*_2_ elements of the *t*^th^ row of the matrix whose columns are right-singular vectors of the matrix SVD of matrix [*U*_1_ *U*_2_ ⋯ *U*_*T*_] at time *t* in Ψ; $W_{X}\in R^{N_{1}\times M_{1}}$ is the linear projection matrix of SVD from *M*_1_ meta-gene expression space to *N*_1_ gene expression space in *X,* $W_{U}\in R^{N_{2}\times M_{2}}$ is the linear projection matrix of SVD from *M*_2_ meta-gene expression space to *N*_2_ gene expression space in Ψ), and (.)^*^ is a pseudo-inverse operation; i.e., W^*^W = *I*, where *I* is the identity matrix. **(C)** Effective state space model for meta-genes. The effective state-space model for meta-genes, Eq 5 is obtained by using linear projections *W*_*X*_ and *W*_*U*_ between genes and meta-genes from Eqs 1–4. The effective meta-gene system matrix ${\tilde{A}=W}_{X}^{*}AW_{X}\in R^{M_{1}\times M_{1}}$ captures internal causal interactions among meta-genes in Ω (i.e., the *i*^th^, *j*^th^ element of $\tilde{A}$, ${\tilde{A}}_{ij}$ describes the contribution from the *j*^th^ meta-gene expression at time *t* to *i*^th^ meta-gene expression at next time *t*+1), and the effective control matrix ${\tilde{B}=W}_{X}^{*}BW_{U}\in R^{M_{1}\times M_{2}}$ captures external causal regulations from meta-genes in Ψ to meta-genes in Ω (i.e., the *i*^th^, *j*^th^ element of $\tilde{B}$, ${\tilde{B}}_{ij}$ describes the contribution from the *j*^th^ meta-gene expression in Ψ at time *t* to *i*^th^ meta-gene expression in Ω at next time *t*+1). Eq 5 describes the effective state space model for the meta-genes in Ω, whose expression dynamics are determined by $\tilde{A}$ and $\tilde{B}$. Because the meta-gene dimension, *M*_1_ (*M*_2_) is less than *T*, and much less than *N*_1_ (*N*_2_), we can estimate $\tilde{A}$ and $\tilde{B}$.

A gene regulatory network is made up of various subsystems [1,2]. These subsystems work together to execute regulatory functions. Given a group of *N*_1_ genes in a subsystem, defined as the internal gene set, Ω, their gene expression levels are not only controlled by internal interactions among Ω, but also affected by the regulatory factors from other subsystems outside Ω. We define an external gene set, Ψ consisting of those external regulatory factors. For example, we consider the worm-fly orthologous genes as internal set Ω. The worm-fly orthologous TFs from internal set Ω are the *internal* regulatory factors, and non-orthologous TFs such as worm- or fly- specific TFs are the *external* regulatory factors. Both the internal and external regulatory factors control gene expressions in dynamic ways (i.e., their regulatory signals at the current time will affect gene expressions at subsequent times). Thus, the regulatory mechanisms for gene expressions form a control system. In this study, we used a state-space model (defined by linear first-order difference equations, Fig 2A) to formulate temporal gene expression dynamics for internal set Ω (comprising *N*_1_ genes) with external regulation from external set Ψ (comprising *N*_2_ genes) at time points *1*, *2*, …, *T* as follows:

$$X_{t+1}=AX_{t}+BU_{t}$$ (1),

where the vector $X_{t}\in R^{N_{1}\times 1}$, the “state”, includes *N*_1_ gene expression levels at time *t* in Ω, and the vector $U_{t}\in R^{N_{2}\times 1}$, the “input or control”, includes *N*_2_ gene expression levels at time *t* in Ψ. The system matrix $A\in R^{N_{1}\times N_{1}}$ captures internal causal interactions among genes in Ω (i.e., the *i*^th^, *j*^th^ element of *A*, *A*_*ij*_ describes the contribution from the *j*^th^ gene expression at time *t* to the *i*^th^ gene expression at the next time *t*+1), which instantiates a gene regulatory network. The control matrix $B\in R^{N_{1}\times N_{2}}$ captures external causal regulations from the genes in Ψ to genes in Ω (i.e., the *i*^th^, *j*^th^ element of *B*, *B*_*ij*_ describes the contribution from the *j*^th^ gene expression in Ψ at time *t* to the *i*^th^ gene expression in Ω at the next time *t*+1). R represents the real number domain. According to the state space model (1), the gene expression dynamics in Ω is determined by the system matrix *A* and the control matrix *B*. In particular, based on Eq 1, the state *X*_*t*_ can be expanded as follows:

$$\begin{array}{l} X_{t}=AX_{t-1}+BU_{t-1}=A(AX_{t-2}+BU_{t-2})+BU_{t-1}=A^{2}X_{t-2}+ABU_{t-2}+BU_{t-1}=A^{3}X_{t-3}+\\ A^{2}{BU}_{t-3}+ABU_{t-2}+BU_{t-1}=\cdots =A^{t-1}X_{1}+A^{t-2}{BU}_{1}+A^{t-3}{BU}_{2}+\cdots +ABU_{t-2}+BU_{t-1}=\\ \underset{X_{t}^{\text{INT}}}{\underbrace{A^{t-1}X_{1}}}+\underset{X_{t}^{\text{INTER}}}{\underbrace{\sum_{k=1}^{t-2}A^{k}BU_{t-1-k}}}+\underset{X_{t}^{\text{EXT}}}{\underbrace{BU_{t-1}}} \end{array}$$ (2),

where $X_{t}^{INT}=A^{t-1}X_{1}$ is defined as the expression vector of the gene components driven only internally by genes in Ω. The rest terms $\sum_{k=1}^{t-2}A^{k}BU_{t-1-k}+BU_{t-1}$ captures the expression expression vector of the gene components in Ω affected externally by the genes in Ψ. In particular, $X_{t}^{\text{EXT}}=BU_{t-1}$ represents the expression vector of gene components in Ω driven purely by the genes in Ψ since it only involves *B* and *U*, and $X_{t}^{\text{INTER}}=\sum_{k=1}^{t-2}A^{k}BU_{t-1-k}$ captures the expression vector of gene components in Ω driven by the interactions between internal and external groups for involving *A*, *B* and *U*. In this paper, we mainly focus on the purely internal dynamics. As for the external-related terms, we should emphasize that any subdivision of the rest of the terms $\sum_{k=1}^{t-2}A^{k}BU_{t-1-k}+BU_{t-1}$ is completely arbitrary. That is, although we subdivided it into a purely external term and an interaction term here, one could subdivide it multiple ways. That is, given a particular type of subdivision, each of the subdivided terms sums up a group of terms from *A*^*k*^*BU*_*t*−1−*k*_, *k* = 0,1,2,…,*t*-2. For example, one can look at $\sum_{k=2}^{t-2}A^{k}BU_{t-1-k}+(ABU_{t-2}+BU_{t-1})$, where *ABU*_*t*−2_ + *BU*_*t*−1_ shows the contribution from the inputs up to two time steps back to *X*_*t*_.

### Dimensionality reduction from genes to meta-genes

The temporal gene expression experiments normally have limited time samples (for example, there may only be a dozen time points), which are far less than the time samples needed to estimate the large matrices *A* and *B* when internal and external groups, Ω and Ψ are composed of hundreds or thousands of genes. One way to deal with lack of time samples is dimensionality reduction. Thus, we project high dimensional temporal gene expressions to much lower dimensional meta-gene expression levels using a dimensionality reduction technique (Fig 2B). Those meta-gene expression levels should capture original gene expression patterns, such as the ones having the greatest degree of co-variation. We calculate the meta-gene expression levels as follows:

$${\tilde{X}}_{t}=W_{X}^{*}X_{t};{\tilde{U}}_{t}=W_{U}^{*}U_{t}$$ (3),

where ${\tilde{X}}_{t}\in R^{M_{1}\times 1}$, the “meta-gene state” at time *t*, includes *M*_1_ (<< *N*_1_ and <*T*) meta-gene expression levels; i.e., the first *M*_1_ elements of the *t*^th^ row of the matrix whose columns are right-singular vectors of the matrix [*X*_1_ *X*_2_ ⋯ *X*_*T*_] in Ω by the singular value decomposition (SVD) [19]; the vector ${\tilde{U}}_{t}\in R^{M_{2}\times 1}$, the “meta-gene input or control” at time *t*, includes *M*_2_ (<< *N*_2_ and <*T*) meta-gene expression levels; i.e., the first *M*_2_ elements of the *t*^th^ row of the matrix whose columns are right-singular vectors from SVD of the matrix [*U*_1_ *U*_2_ ⋯ *U*_*T*_] in Ψ; $W_{X}\in R^{N_{1}\times M_{1}}$ is the linear projection matrix of SVD from *M*_1_ meta-gene expression space to *N*_1_ gene expression space in Ω, $W_{U}\in R^{N_{2}\times M_{2}}$ is the linear projection matrix of SVD from *M*_2_ meta-gene expression space to *N*_2_ gene expression space in Ψ, and (.)^*^ is a pseudo-inverse operation; i.e., W^*^W = *I*, where *I* is the identity matrix.

### Estimation of effective state-space model for meta-gene expression dynamics

Next, we obtain the effective state-space model for meta-genes using linear projections *W*_*X*_ and *W*_*U*_ between genes and meta-genes as follows (Fig 2C). By replacing (1) using (3), we obtain that

$$W_{X}{\tilde{X}}_{t+1}=AW_{X}{\tilde{X}}_{t}+BW_{U}{\tilde{U}}_{t}.$$ (4)

After multiplying the pseudo-inverse of *W*_*X*_, $W_{X}^{*}\in R^{M_{1}\times N_{1}}$ s.t. $W_{X}^{*}W_{X}=I$ where *I* is an identity matrix, at both sides of (4), we have that

$${\tilde{X}}_{t+1}=\underset{\tilde{A}}{\underbrace{W_{X}^{*}AW_{X}}}\;{\tilde{X}}_{t}+\underset{\tilde{B}}{\underbrace{W_{X}^{*}BW_{U}}}\;{\tilde{U}}_{t}=\tilde{A}{\tilde{X}}_{t}+\tilde{B}{\tilde{U}}_{t}$$ (5),

where the effective meta-gene system matrix ${\tilde{A}=W}_{X}^{*}AW_{X}\in R^{M_{1}\times M_{1}}$ captures internal causal interactions among meta-genes in Ω (i.e., an element of $\tilde{A}$, ${\tilde{A}}_{ij}$ describes the contribution from the *j*^th^ meta-gene expression at time *t* to *i*^th^ meta-gene expression at time *t*+1), and the effective control matrix ${\tilde{B}=W}_{X}^{*}BW_{U}\in R^{M_{1}\times M_{2}}$ captures external causal regulations from meta-genes of Ψ to meta-genes of Ω (i.e., the *i*^th^, *j*^th^ element of $\tilde{B}$, ${\tilde{B}}_{ij}$ describes the contribution from the *j*^th^ meta-gene expression in Ψ at time *t* to *i*^th^ meta-gene expression in Ω at time *t*+1). Eq 5 describes the effective state space model for the meta-genes of Ω, whose expression dynamics is determined by $\tilde{A}$ and $\tilde{B}$. Because the meta-gene dimension, *M*_1_ (*M*_2_) is less than *T*, and much less than *N*_1_ (*N*_2_), we can estimate $\tilde{A}$ and $\tilde{B}$ as follows.

We rewrite Eq 5 as a matrix product on the right side:

$${\tilde{X}}_{t+1}=\tilde{A}{\tilde{X}}_{t}+\tilde{B}{\tilde{U}}_{t}=[\begin{array}{cc} \tilde{A} & \tilde{B} \end{array}]\left[\begin{array}{c} {\tilde{X}}_{t}\\ {\tilde{U}}_{t} \end{array}\right].$$ (6)

By applying Eq 6 to time points, 2,3, …, *T*, we then obtain that

$$\underset{Ζ}{\underbrace{\left[\begin{array}{ccc} \begin{array}{cc} {\tilde{X}}_{2} & {\tilde{X}}_{3} \end{array} & \cdots & {\tilde{X}}_{T} \end{array}\right]}}=[\begin{array}{cc} \tilde{A} & \tilde{B} \end{array}]\underset{\Upsilon }{\underbrace{\left[\begin{array}{ccc} \begin{array}{cc} {\tilde{X}}_{1} & {\tilde{X}}_{2} \end{array} & \cdots & {\tilde{X}}_{T-1}\\ \begin{array}{cc} {\tilde{U}}_{1} & {\tilde{U}}_{2} \end{array} & \cdots & {\tilde{U}}_{T-1} \end{array}\right]}}$$ (7),

where $Ζ\in R^{M_{1}\times (T-1)}$ and $\Upsilon \in R^{{(M}_{1}+M_{2})\times (T-1)}$.

Because of dimension reduction, Υ has more columns than rows so that it has right pseudo-inverse. Thus, the effective internal system matrix $\tilde{A}$ and external control matrix $\tilde{B}$ can be estimated by:

$$[\begin{array}{cc} \tilde{A} & \tilde{B} \end{array}]=Ζ\Upsilon^{*}$$ (8),

where $\Upsilon^{*}\in R^{\left(T-1)\times {(M}_{1}+M_{2}\right)}$ is the right pseudo-inverse of Υ; i.e., ΥΥ* = *I*, with *M*_1_<*N*_1_, *M*_2_<*N*_2_, *M*_1_+*M*_2_<*T*, *t* = 1,2,…,*T*. It is worth noting that if we do not reduce the dimensionality, and obtain Eq 7 from Eq 5, then Υ will have much more rows than columns so that it doesn’t have right pseudo-inverse; i.e., there doesn’t exist a matrix Υ* such that ΥΥ* is a full-rank identify matrix. In addition, the condition of *M*_1_+*M*_2_<*T* also makes ΥΥ* be a full-rank identify matrix.

### Identification of internally and externally driven principal dynamic expression patterns of meta-genes (canonical temporal expression trajectories)

The analytic solution to a general first-order linear matrix difference equation [20], *Q*_*t*+1_ = *CQ*_*t*_ is

*Q*_*t*_ = *C*^*t*^*Q*_*0*_ *= (HEH*^*-1*^*)* ^*t*^*Q*_*0*_ *= HE*^*t*^*H*^-1^*Q*_*0*_ *= HE*^*t*^*S*, where the columns of the matrix *H* are eigenvectors of *C*, the diagonal elements of the diagonal matrix *E* are eigenvalues of *C* such that *CH* = *HE*, and the vector

*S* = *H*^-1^*Q*_*0*_. Then, if we rewrite *Q*_*t*_ by a linear combination of the time exponential of eigenvalues of *C*, we have that $Q_{t}=HE^{t}S=\sum_{i=1}^{m_{c}}\alpha_{i}^{t}s_{i}H_{i}=\sum_{i=1}^{m_{c}}\alpha_{i}^{t}K_{i}$, where *m*_*c*_ is the total number of eigenvalues of *C*, *α*_*i*_ is the *i*^th^ eigenvalue of *C*, *s*_*i*_ is the *i*^th^ element of *S*, *H*_*i*_ is the *i*^th^ eigenvector of *C* (i.e., the *i*^th^ column of *H*), and *K*_*i*_ = *s*_*i*_*H*_*i*_ is the coefficient vector of *Q*_*t*_ over the *t*^th^ time exponential of *α*_*i*_.

By Eq 5, the matrix *Ã* determines the meta-gene states components whose expression dynamics are internally controlled by the meta-genes of Ω. As Eq 2, we define the expression of the meta-gene components driven only internally by themselves in Ω at time *t* as ${\tilde{X}}_{t}^{INT}$, an *M*_1_-dimensional vector; i.e., their expression at two adjacent time points have ${\tilde{X}}_{t+1}^{INT}=\tilde{A}{\tilde{X}}_{t}^{INT}\in R^{M_{1}\times 1}$. According to the above analytic solution, it can be a linear combination of *M*_1_ dynamic patterns determined by the eigenvalues of the effective system matrix $\tilde{A}$ as follows:

${\tilde{X}}_{t}^{INT}=\sum_{p=1}^{M_{1}}\lambda_{p}^{t}{\tilde{K}}_{p}$; i.e., the internally driven component of *i*^th^ meta-gene’s expression across all time points,

$$[\begin{array}{cc} \begin{array}{cc} {\tilde{X}}_{1}^{INT}(i) & {\tilde{X}}_{2}^{INT}(i) \end{array} & \begin{array}{cc} \ldots & {\tilde{X}}_{T}^{INT}(i) \end{array} \end{array}]=\sum_{p=1}^{M_{1}}{\tilde{K}}_{p}(i)\underset{p^{th}\;iPDP}{\underbrace{\left[\begin{array}{cc} \begin{array}{cc} \lambda_{p}^{1} & \lambda_{p}^{2} \end{array} & \begin{array}{cc} \ldots & \lambda_{p}^{T} \end{array} \end{array}\right]}}$$ (9),

where *λ*_*p*_ and ${\tilde{K}}_{p}\in C^{M_{1}\times 1}$ are the *p*^th^ eigenvalue of $\tilde{A}$ and its coefficient vector from the analytic solution, which determines the *p*^th^ dynamic pattern driven by effective internal regulations, defined as the *p*^th^ internal principal dynamic pattern (iPDP) = $\left[\lambda_{p}^{1}\;\lambda_{p}^{2}\;\ldots \;\lambda_{p}^{T}\right]$, in which $\lambda_{p}^{t}$ represents the *t*^th^ power of *λ*_*p*_, and *Ξ*(*i*) represents *i*^th^ element of the vector *Ξ*. C represents the complex number domain. If an eigenvalue *λ* is complex when $\tilde{A}$ is asymmetric, then its conjugate $\bar{\lambda }$ is also an eigenvalue, so we sum its iPDP and its conjugate eigenvalue, $\bar{\lambda }$’s iPDP, as a unified iPDP with real elements equal to $\left[\lambda_{p}^{1}{+\bar{\lambda }}_{p}^{1}\;\lambda_{p}^{2}{+\bar{\lambda }}_{p}^{2}\;\ldots \;\lambda_{p}^{T}{+\bar{\lambda }}_{p}^{T}\right]$.

**Fig 3:**
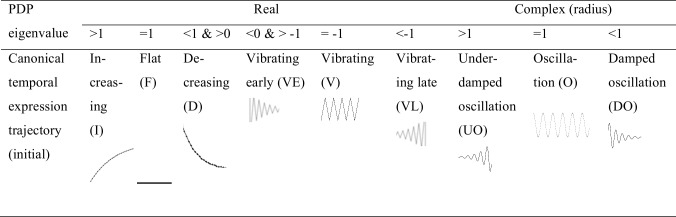
Classification of canonical temporal expression trajectories for iPDP eigenvalue types. The internal principal dynamic patterns (iPDPs) represent canonical temporal expression trajectories, which can be either increasing, or damped oscillation and so on depending on iPDP’s eigenvalues (The bottom row).

The internal principal dynamic patterns (iPDPs) represent canonical temporal expression trajectories, which can be either increasing, or damped oscillation and so on depending on iPDP’s eigenvalues (Fig 3). The iPDPs can be ordered by sorting their eigenvalues.

Also by Eqs 2 and 5, the expression of the meta-gene states components driven purely by the external group Ψ at time *t* is defined as ${\tilde{X}}_{t}^{EXT}$, an *M*_1_-dimensional vector, and its expression dynamics is determined by the equation ${\tilde{X}}_{t+1}^{EXT}=\tilde{B}{\tilde{U}}_{t}\in R^{M_{1}\times 1}$; i.e., the externally driven components of meta-gene states at two adjacent time points. In particular, the externally driven component of *i*^th^ internal meta-gene’s expression across time points:

$$[\begin{array}{cc} \begin{array}{cc} {\tilde{X}}_{2}^{EXT}(i) & {\tilde{X}}_{3}^{EXT}(i) \end{array} & \begin{array}{cc} \ldots & {\tilde{X}}_{T}^{EXT}(i) \end{array} \end{array}]=\sum_{q=1}^{M_{2}}{\tilde{B}}_{i,q}\;\underset{q^{th}\;ePDP}{\underbrace{\left[\begin{array}{cc} \begin{array}{cc} {\tilde{U}}_{1}(q) & {\tilde{U}}_{2}(q) \end{array} & \begin{array}{cc} \ldots & {\tilde{U}}_{T-1}(q) \end{array} \end{array}\right]}}$$ (10),

where ${\tilde{X}}_{t}^{EXT}(i)$ and ${\tilde{U}}_{t}(q)$ are *i*^th^ and *q*^th^ elements of ${\tilde{X}}_{t}^{EXT}$ and ${\tilde{U}}_{t}$, respectively with *t* = 1,2,…, *T*, the vector $\underbrace{\left[\begin{array}{cc} \begin{array}{cc} {\tilde{U}}_{1}(q) & {\tilde{U}}_{2}(q) \end{array} & \begin{array}{cc} \ldots & {\tilde{U}}_{T-1}(q) \end{array} \end{array}\right]}$ is defined as *q*^th^ external principal dynamic pattern (ePDP), and ${\tilde{B}}_{i,q}$ is the element of $\tilde{B}$ at *i*^th^ row and *q*^th^ column, which is also the coefficient of the externally driven component of *i*^th^ internal meta-gene’s expression over *q*^th^ ePDP. Based on Eq 2, the expression of the meta-gene components driven by the interactions between internal and external meta-genes is given by ${\tilde{X}}_{t}^{\text{INTER}}=\sum_{k=1}^{t-2}{\tilde{A}}^{k}\;\tilde{B}{\tilde{U}}_{t-1-k}.$ In this paper, we focus on the purely driven internal patterns (i.e., iPDPs) and compare them across different biological systems.

### Identification of gene coefficients of principal expression dynamic patterns

Because genes and meta-genes have linear relationships in terms of their expression levels as described in Eq 2, the components of gene expression levels in Ω driven by internal regulations, $X_{t}^{INT}\in R^{N_{1}\times 1}$ can be also expressed as linear combinations of *M*_1_ iPDPs:

$${{X_{t}^{INT}=W}_{X}\;\tilde{X}}_{t}^{INT}=\sum_{p=1}^{M_{1}}\;\lambda_{p}^{t}\;\underset{C_{p}}{\underbrace{W_{X}\;{\tilde{K}}_{p}}}=\sum_{p=1}^{M_{1}}\;\lambda_{p}^{t}\;C_{p}\;;\;i.e.,$$

the internally driven component of *i*^th^ gene’s expression across all time points,

$$[\begin{array}{cc} \begin{array}{cc} X_{1}^{INT}(i) & X_{2}^{INT}(i) \end{array} & \begin{array}{cc} \ldots & X_{T}^{INT}(i) \end{array} \end{array}]=\sum_{p=1}^{M_{1}}C_{p}(i)\;\underset{p^{th}\;iPDP}{\underbrace{\left[\begin{array}{cc} \begin{array}{cc} \lambda_{p}^{1} & \lambda_{p}^{2} \end{array} & \begin{array}{cc} \ldots & \lambda_{p}^{T} \end{array} \end{array}\right]}}$$ (11),

where $C_{p}=W_{X}\;{\tilde{K}}_{p}\in C^{M_{1}\times 1}$ represents the gene coefficient vector for *p*^th^ iPDP. Similarly, the gene expression components driven by external genes in Ψ, $X_{t}^{EXT}\in R^{N_{1}\times 1}$ can be also expressed as linear combinations of *M*_2_ ePDPs:

$$X_{t}^{EXT}={W_{X}\;\tilde{X}}_{t}^{EXT}=\underset{D}{\underbrace{W_{X}\;\tilde{B}}}\;{\tilde{U}}_{t}=D{\tilde{U}}_{t}\;;\;i.e.,$$

the externally driven component of *i*^th^ gene’s expression across all time points,

$$[\begin{array}{cc} \begin{array}{cc} X_{2}^{EXT}(i) & X_{3}^{EXT}(i) \end{array} & \begin{array}{cc} \ldots & X_{T}^{EXT}(i) \end{array} \end{array}]=\sum_{q=1}^{M_{2}}D_{i,q}\;\underset{q^{th}\;ePDP}{\underbrace{\left[\begin{array}{cc} \begin{array}{cc} {\tilde{U}}_{1}(q) & {\tilde{U}}_{2}(q) \end{array} & \begin{array}{cc} \ldots & {\tilde{U}}_{T-1}(q) \end{array} \end{array}\right]}}$$ (12),

where $X_{t}^{EXT}(i)$ is *i*^th^ element of $X_{t}^{EXT}$ with *t* = 1,2,…, *T*, and *D*_*i*,*q*_ is the element of $D=W_{X}\;\tilde{B}$ at *i*^th^ row and *q*^th^ column, which is also the coefficient of the externally driven component of *i*^th^ gene’s expression over *q*^th^ ePDP.

## Results

Gene expression data during embryogenesis provide important information about the dynamics of genomic functions throughout the developmental process, from the conserved functions such as DNA replication to the species-specific functions such as body segmentation, but hardly reveal any data regarding the evolutionary gene regulatory subsystems that drive those developmental functions [3]. Thus, in order to understand the relationships between those subsystems and their driving genomic functions, we apply DREISS to worm and fly gene expression datasets during embryogenesis in modENCODE and we are able to identify various developmental genomic functions of worm-fly orthologous gene pairs driven by two different evolutionary regulatory subsystems, conserved (worm-fly orthologous TFs) and non-conserved (worm/fly specific TFs). As model organisms for developmental biology, both worm and fly have been used previously to study embryogenesis.

### Applications to worm and fly embryonic developmental data in modENCODE: Orthologous genes, transcription factors and gene expression datasets

DREISS enables us to compare expression dynamic patterns between two or more temporal gene expression datasets even though they have different numbers of samples, as well as differences in the times at which those samples were collected. For example, we can apply DREISS to two different datasets of the same group of genes, and identify both the common (similar) and the specific (different) dynamic patterns driven by internal regulations captured by the eigenvalues of the effective system matrices between the two datasets.

In this paper, we apply DREISS to 3,153 one-to-one orthologous genes between worm (*Caenorhabditis elegans*) and fly (*Drosophila melanogaster*) as internal group, Ω to study their expression dynamics during embryonic development [10]. We refer to species-specific TFs as external regulations; i.e., external group Ψ. We found that worm-fly orthologs have similar internal dynamic patterns, which may be mainly driven by conserved TFs, but have very different external dynamic patterns driven by species-specific TFs between worm and fly embryonic developmental stages. The data is summarized as follows.

We define internal group Ω as 3,153 one-to-one orthologous genes between worm and fly during embryonic development, and external group Ψ as all the species-specific TFs (509 worm-specific TFs, 442 fly-specific TFs) [21,22]. We used their temporal gene expression levels (as measured by the RPKM values in RNA-seq) during embryonic development from the modENCODE project [10]. The worm embryonic development dataset includes *T* = 25 time stages at 0, 0.5, 1, 1.5, …, 12 hours, and the fly dataset includes *T* = 12 time stages at 0, 2, 4, …, 22 hours, but *t* = 1,2,..,25 for worm and *t* = 1,2,…,12 for fly are used in this paper, representing the relative time points for the entire embryonic development processes. Because *M*_1_+ *M*_2_<*T* in Eq 8, we choose *M*_1_ = *M*_2_ = 5 meta-genes for fly (*T* = 12), and find that five meta-genes of Ω and five meta-genes of Ψ capture ~98% of the co-variation of orthologous gene expressions and fly-specific TF gene expressions, respectively. In order to compare worm and fly, we also choose *M*_1_ = *M*_2_ = 5 meta-genes for worm, which capture ~98% of the co-variation of orthologous gene expressions and worm-specific TF gene expressions.

### Meta-genes of worm-fly orthologous genes have similar internal, yet different external principal dynamic patterns during embryonic development

**Fig 4:**
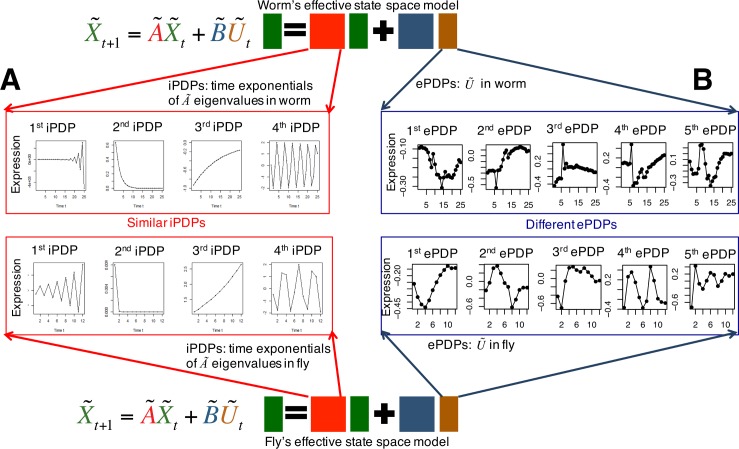
Principal dynamic patterns of orthologous genes between worm and fly during embryonic development. **(A)** Metagenes of orthologous genes have similar internal driven principal dynamic patterns. Meta-gene canonical temporal expression trajectories driven by conserved regulatory networks (i.e., internal principal dynamic patterns, iPDPs) include four major patterns in both worm and fly embryonic development: 1) a highly varied pattern late (iPDP with the real eigenvalue No. 1); 2) a fast decaying pattern early (iPDP with the real eigenvalue No. 2); 3) a slowly increasing pattern (iPDP with the real eigenvalue No. 3); and 4) an oscillating pattern (iPDP with the complex eigenvalue). **(B)** Metagenes of orthologous genes have different external driven principal dynamic patterns. Worm and fly have very different external principal dynamic patterns (ePDPs); i.e., the patterns driven by species-specific TFs. The principal dynamic patterns driven by the worm-specific regulatory network; i.e., worm ePDPs consist of a varied pattern that decreases until the middle stage and then increases (ePDP No.1), an increasing pattern (ePDP No.2), a varied pattern with a peak entering middle stage ((ePDP No.3), a pattern that varies early and then increases during the embryonic development (ePDP No.4), and a cosine-like oscillating pattern with roughly two periods during the embryonic development (ePDP No.5). The fly ePDPs, however, have a varied pattern with low expression at the early stage (ePDP No.1), a sine-like oscillating pattern with roughly one period during the embryonic development (ePDP No.2), an increasing pattern (ePDP No.3), another sine-lie oscillating pattern with roughly two periods during the embryonic development (ePDP No.4), and a varied pattern that is like damped oscillation (ePDP No.5).

We find that the meta-gene canonical temporal expression trajectories driven by conserved regulatory networks (i.e., internal principal dynamic patterns, iPDPs) include four major patterns in both the worm and fly embryonic developmental process by order of eigenvalues: 1) a late highly varied pattern; 2) an early fast decaying pattern; 3) a slowly increasing pattern; and 4) an oscillating pattern (Fig 4A); i.e., the pattern of canonical trajectories (VL, D, I, O) in Fig 3. In contrast to the observed iPDP similarities, we find that worm and fly have very different external principal dynamic patterns (ePDPs) (Fig 4B); i.e., the expression dynamic patterns driven by species-specific TFs. The principal dynamic patterns driven by the worm-specific regulatory network; i.e., worm ePDPs, include a varied pattern that decreases until the middle stage and then increases, an increasing pattern, a varied pattern with a peak entering middle stage, a pattern that varies early and then increases during the embryonic development, and a cosine-like oscillating pattern with roughly two periods during the embryonic development. The fly ePDPs, however, have a varied pattern with low expression at the early stage, a sine-like oscillating pattern with roughly one period during the embryonic development, an increasing pattern, another sine-lie oscillating pattern with roughly two periods during the embryonic development, and a varied pattern that is like damped oscillation. In addition, we checked the sensitivity of iPDPs to small perturbations to internal/external regulatory networks by the leave-one-out method; i.e., we removed one gene in the internal/external group, ran DREISS, and obtained the ordered iPDP eigenvalues for the remaining genes. We repeated the leave-one-out method for all genes, and finally found the ranges in which iPDP eigenvalues vary shown as error bars in S1 Fig. We can see that the iPDP eigenvalues almost stay at the same values (small error bars) for both worm and fly, which implies that the principal dynamic patterns of worm-fly orthologous genes driven by their conserved regulatory network are robust to small changes.

The above results suggest that the conserved regulatory networks from orthologous meta-genes between worm and fly have similar effects to orthologous meta-genes, given their similar iPDPs (i.e., both have four patterns, as described above). The species-specific regulatory networks from species-specific meta-genes (i.e., worm-specific or fly specific TFs) have effects that differ from the orthologous meta-genes for their different ePDPs. In addition, the expression dynamic patterns driven by the interactions between internal orthologous genes and external species-specific TFs are also different between worm and fly (S2 Fig).

### Orthologous genes have correlated coefficients between worm and fly for their matched internal principal dynamic patterns

**Fig 5:**
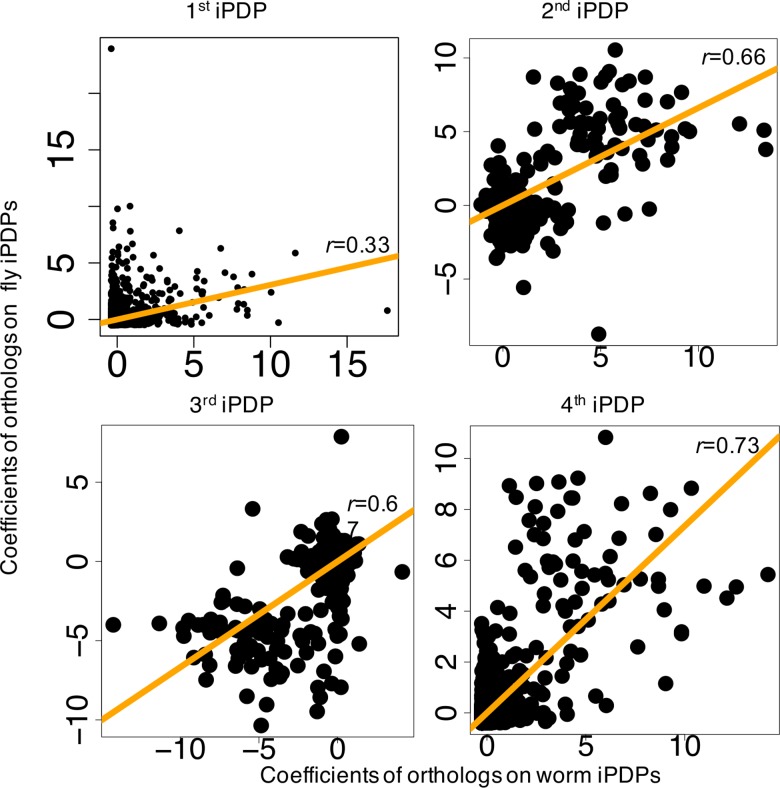
Orthologous genes have correlated coefficients between worm and fly for their matched internal principal dynamic patterns. The 3,153 worm-fly orthologous genes have correlated coefficients over each of four iPDPs. Their coefficients are significantly correlated between worm and fly iPDPs with a similar pattern: r = 0.33 (*p*<2.2e-16) for the highly varied pattern at late embryonic development (first iPDP), r = 0.66 (*p*<2.2e-16) for the fast decaying pattern at early embryonic development (second iPDP), r = 0.67 (*p*<2.2e-16) for the slowly increasing pattern during embryonic development (third iPDP), and r = 0.73 (*p*<2.2e-16) for the oscillation pattern during embryonic development (forth iPDP).

In both worm and fly, we observe the similar four types of internally driven canonical temporal expression trajectories; i.e., four matched internal principal dynamic patterns (iPDPs) (Fig 4A). Thus, we are interested in seeing how individual orthologous genes relate to those dynamic patterns. We find that the worm-fly orthologous genes have correlated coefficients over each of the four iPDPs. Based on Eq 10, we can obtain the coefficients of orthologous genes for each iPDP. We find that their coefficients are significantly correlated between worm and fly iPDPs with a similar pattern (Fig 5): *r* = 0.33 (*p*<2.2e-16) for the highly varied pattern at late embryonic development stages (first iPDP), *r* = 0.66 (*p*<2.2e-16) for the fast decaying pattern at early embryonic development stages (second iPDP), *r* = 0.67 (*p*<2.2e-16) for the slowly increasing pattern during embryonic development (third iPDP), and *r* = 0.73 (*p*<2.2e-16) for the oscillation pattern during embryonic development (forth iPDP), where *r* represents Spearman correlation of iPDP coefficients of 3,153 orthologous genes between worm and fly. This implies that, not only do the orthologous meta-genes have similar internal (conserved) regulatory effects (i.e., similar iPDPs), but the worm-fly orthologous genes also have similar internally-driven expression dynamics as resulted from their significantly correlated coefficients for iPDPs. The ePDPs between worm and fly generally do not show a high degree of matching similarity, but the worm ePDP No. 2, and the fly ePDPs No. 3 are roughly representing the growing patterns. We find that orthologous gene correlation coefficients between these ePDP patterns are very small (Spearman correlation *r* = -0.22 of the orthologous gene coefficients of worm ePDP No.2 and fly ePDP No. 3).

### Ribosomal genes have significantly larger coefficients for the internal than external principal dynamic patterns, but signaling genes exhibit the opposite trend

**Fig 6:**
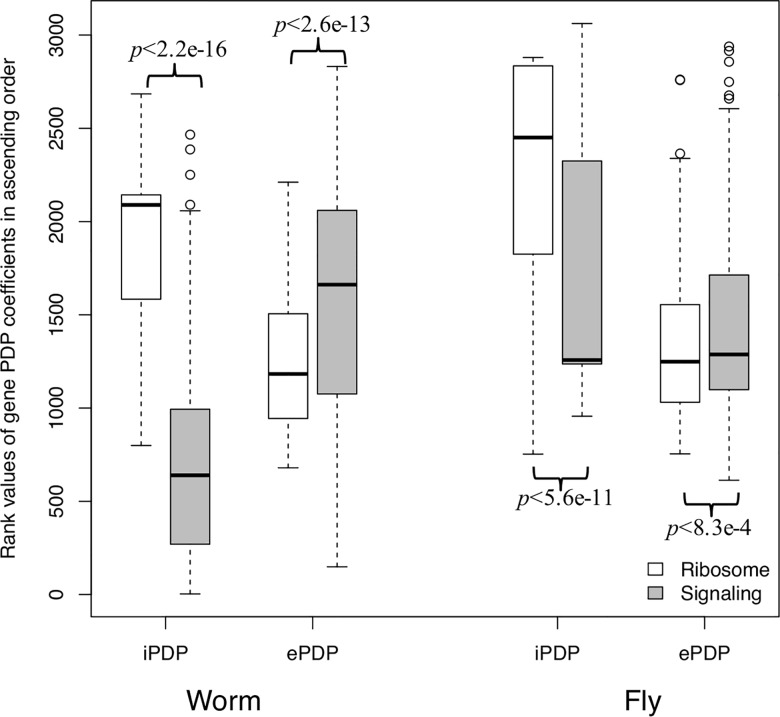
Ribosomal genes have significantly larger coefficients for internal than external principal dynamic patterns, but signaling genes exhibit the opposite trend. The rank values in ascending order of iPDP and ePDP coefficients of ribosomal and signaling genes (cell-cell communication) genes are compared. The y-axis shows the distributions of rank values. Ribosomal genes (white boxes): their average rank values of iPDP coefficients are significantly larger than ePDP ones in both worm (*t*-test *p*<2.2e-16) and fly (*t*-test *p*<5.6e-11). Signaling genes (grey boxes): they have significantly larger average rank values of ePDP coefficients than iPDP ones in both worm (*t*-test p<2.6e-13) and fly (*t*-test p<8.3e-4).

The ribosome produces proteins, which is an ancient process and conserved across worm and fly, organisms separated by almost a billion years of evolution. The ribosomal genes are highly expressed during embryogenesis, since intensive cell division and migration require a large amount of proteins to be synthesized. We collected 195 ribosome-related genes based on the GO annotations. We ranked the coefficients of orthologous genes for each iPDP and ePDP in ascending order, and compared the rank values of iPDP and ePDP coefficients of ribosomal genes. We found that their average ranks of iPDP coefficients are significantly larger than ePDP ones in both worm (*t*-test *p*<2.2e-16) and fly (*t*-test *p*<2.6e-13) as shown in Fig 6. This means that the ribosomal gene expression is significantly more influenced by the conserved regulatory network than by the species-specific regulatory network, which is consistent with ribosomal genes having conserved functions during embryonic development.

The orthologous genes related to signal transduction for cell-cell communication (a significantly more recent evolutionary adaptation relative to the ribosome) exhibit the opposite trend. We found that 320 signaling genes from GO annotations have significantly larger average rank values of ePDP coefficients than iPDP ones in both worm (*t*-test *p*<5.6e-11) and fly (*t*-test *p*<8.3e-4), as shown in Fig 6. This result implies that the signaling gene expression is significantly more driven by the species-specific regulatory network than by the conserved regulatory network, which is consistent with the signaling genes being commonly associated with species-specific functions, such as body plan establishment and cell differentiation.

### DNA replication and Proteasome machinery are enriched in orthologous genes with high coefficients for the dynamic patterns with fast growing canonical trajectories

**Fig 7:**
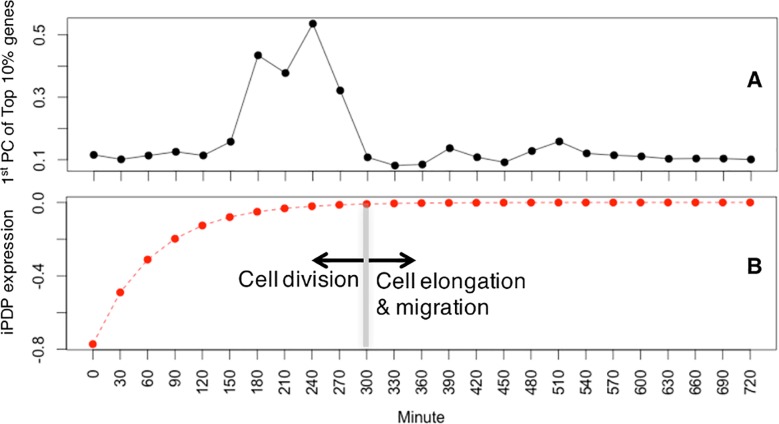
DNA replication is enriched in orthologous genes with high coefficients for the dynamic patterns with fast growing canonical trajectories. **(A)** The first principal component of Top 10% genes with most negative coefficients with 2^nd^ worm iPDP (black curve). **(B)** The fast-growing iPDP (vertical flipped 2^nd^ iPDP) showing a drastic increase at the beginning of embryogenesis, then remain flat during the late embryogenesis (red curve). For the fast-decaying pattern (2^nd^ iPDP), we found that the DNA replication is significantly enriched in Top 300 (~10%) orthologous genes that have the most negative coefficients for this pattern, in both worm (*p*<1.6e-8) and fly (*p*<4.5e-6). The very negative coefficients for the fast decaying pattern means high positive coefficients for a fast-growing pattern (red curve). The original expression patterns of those top orthologous genes actually do not have fast-growing patterns (black curve).

We next turn to the biological meaning of individual canonical temporal expression trajectory for iPDPs and ePDPs. For the fast-decaying pattern (2^nd^ iPDP), we find that the DNA replication is significantly enriched in Top 300 (~10%) orthologous genes that have the most negative coefficients for this pattern, in both worm (*p*<1.6e-8) and fly (*p*<4.5e-6). The GO enrichment analysis was performed using DAVID [23]. The very negative coefficients for the fast decaying pattern mean high positive coefficients for a fast-growing pattern (vertically flipped 2^nd^ iPDPs of worm and fly represent a fast-growing pattern), showing a drastic increase at the beginning of embryogenesis, then remain flat during the late embryogenesis (red curves in Fig 7). Most of the cell division of embryogenesis in both worm and fly happens approximately within the first 300 minutes. Then, the cell elongation and migration start to dominate the development [24,25]. The mRNA abundance of the genes involved in DNA replication may change accordingly. This is well reflected by the second iPDP. Interestingly, the original expression patterns of those top orthologous genes actually do not have fast-growing patterns (black curves in Fig 7), probably because of the combined effects of both conserved and species-specific GRN. Maternal mRNAs, which are pre-loaded before fertilization, may also mask the fast growing pattern of DNA replication genes. This pattern could only be observed after we separated the effect of two types of TFs using DREISS. In addition, we did not find any enrichment of DNA replication in top genes of other iPDPs (*p*>0.05). Therefore, the fast-growing iPDP patterns identified by our method reveal conserved regulation on the elementary cellular process of both species (i.e. DNA replication).

Besides a fast growing pattern driven by conserved worm-fly orthologous TFs, we also identified a fast growing pattern driven by non-conserved TFs for the two species. The Top 300 orthologous genes (~10%) with the fast-growing worm ePDP (ePDP No.2) (i.e., driven by species-specific regulatory networks) are enriched in ‘proteasome’ (*p*<9.8e-16). Protein degradation is not only a key process in apoptosis, but also throughout the entire course of development [26,27]. For example, eliminating proteins that are no longer needed is a vital process during embryo development; e.g., the maternal proteins need to be cleaned as the embryogenesis proceeds). Previous reports also showed that different species usually have different maternal mRNA in the oocyte, which indicates that species-specific strategies might be utilized to regulate the protein degradation process [28]. In this study, after separating the effect of conserved and non-conserved regulatory networks, we observed that the protein degradation is significantly enriched in the genes majorly driven by species-specific TFs in worms. In contrast, the Top 300 orthologous genes with fast growing fly ePDP3 are enriched in ‘mitotic cell cycle’ (*p*<3.5e-29), ‘translation’ (*p*<1e-30) and ‘mitochondrion’ (*p*<7.7e-20). Those enriched function related to energy generation is probably indicative of the large energy requirement during fly embryogenesis [29], which did not provide the evolutionary conservation of this energy-related gene regulation. Our result reveals that the fly genes associated with respiration are more up-regulated by fly-specific TFs relative to conserved TFs, and that this up-regulation evolved after the separation of worm and fly.

Besides the fast-growing pattern driven by species-specific TFs, we also observed some other interesting patterns. For example, worm ePDP3 displays a dramatic peak about 5 hours after fertilization. Among the Top 300 worm orthologous genes of this pattern, genes involved in synaptic transmission (*p*<5.6e-9) and cell-cell signaling (*p*<1e-7) are over-represented, suggesting that they are transiently activated in this stage of embryogenesis by worm-specific TFs. This observation indicates the gene regulatory network for these genes have evolved after the speciation.

### Human-specific transcription factors respond to hormonal stimulation during breast cancer cell cycle

We applied DREISS to another example (also see supplement) about cancer. We are also interested to identify the gene expression dynamic patterns driven by conserved and human-specific regulatory networks during breast cancer cell cycle. Thus, we applied DREISS to a time-series gene expression data for human estrogen-responsive breast cancer cell line (ZR-75.1) before and after hormonal stimulation, which has 12 time points covering a complete mitotic cell cycle (0–32 hours) of hormonal stimulated cells [30]. The internal group, Ω is defined as a set of cross-species conserved human genes (i.e., 1132 worm-fly-human orthologs including 150 orthologous TFs), and the external group, Ψ consists of 1870 human-specific TFs. As shown in S3 Fig, the internally driven principal dynamic patterns (iPDPs) of conserved human genes include an oscillation trajectory whose period is roughly equal to a full cell cycle (iPDP No. 4), but the externally driven patterns (ePDPs No. 2–4) oscillates more frequently than internal one, which suggests that though the evolutionarily conserved TFs regulate the normal cell cycle, the human specific TFs potentially drive the abnormal cycling behaviors of conserved gene expression responding to the hormonal stimulation.

## Discussion

In this paper, we presented a novel computational method, DREISS, which decomposes time-series expression data of a group of genes into the components driven by the regulatory network inside the group (internal regulatory subsystem), and the components driven by the external regulatory network consisting of regulators outside the group (external regulatory subsystem). DREISS is a general-purpose tool that can be used to study the gene regulatory effects of any interested biological subsystems such as protein-coding transcription factors, micro-RNAs, epigenetic factors and so on. As an illustration, we applied DREISS to the time-series gene expression datasets for worm and fly embryonic developments from the modENCODE project [10], and compared the worm-fly orthologous gene expression dynamic patterns driven by the conserved regulatory network (i.e., regulation effects from orthologous TFs), with the patterns driven by the species-specific regulatory networks (i.e., regulation effects from worm or fly specific TFs). We found that the conserved TFs drive similar genomic functions, but non-conserved TFs drive species-specific functions of orthologous genes between worm and fly, implying that, in addition to having ancient conserved functions, orthologous genes have been regulated by evolutionarily younger GRNs to execute species-specific functions during the evolution. This work can be easily extended to study the regulatory effects from orthologous TFs and species-specific TFs to species-specific genes. For example, one can find the expression dynamic patterns of worm/fly specific genes driven by specific TFs, and identify the genes with strong patterns associated with worm/fly specific functions, such as body formations. To the best of our knowledge, DREISS is the first method to reveal how the evolution of GRNs affects gene expression during embryogenesis.

We emphasize that DREISS is a general-purpose method (a free downloadable R tool available from github.com/gersteinlab/dreiss). Users can define the internal group (Ω) and external group (Ψ) according to their interests. For example, if users want to identify the protein-coding expression patterns driven by miRNAs, they can define miRNAs as an external group and protein-coding genes as an internal group. Additionally, DREISS can be applied to more than two datasets, such as comparing worm, fly and human embryonic stem cell developmental data, and finding their conserved and specific developmental expression patterns. The expression patterns driven by human-specific regulatory factors will potentially help us understand human-specific developmental processes along with the associated human genes.

Due to the limited time samples in gene expression datasets, DREISS uses the simple linear state space model (i.e. the first order linear invariant difference equation) to model the temporal gene expression dynamics, and identify principal temporal dynamic patterns. This model assumes that the gene regulatory networks controlling temporal gene expression dynamics does not change across the entire biological process such as (*A*, *B*) in Eq 1. Thus, based on the analytic analysis, the principal dynamic patterns (PDPs) must follow a small set of canonical temporal trajectories (Fig 3). With the rapidly increasing gene expression data, we can extend DREISS to more advanced models such as switched and hybrid system models, non-linear models [31], which will allow us to study the gene regulatory networks are time varying, and potentially find the more temporal gene expression patterns capturing the more complex gene regulatory activities.

## Supporting Information

S1 Fig Principal dynamic patterns and their eigenvalues. Internal principal dynamic patterns (iPDPs) of orthologs during worm and fly embryonic development. Barplots show the eigenvalues of iPDPs. The error bar for each eigenvalue tells the its variation range. We left one gene out, and calculated eigenvalues for the remaining genes thus obtaining the eigenvalue variations. The curves show the canonical temporal expression trajectories of iPDPs.
(TIF)

S2 FigThe expression dynamic patterns driven by the interactions between worm-fly orthologs and species-specific TFs.The first five singular vectors (>95% covariance in total) of $\left[{\tilde{X}}_{t}^{\text{INTER}},\;t=1,2,3,\ldots ,T\right]$ defined at the end of Section “Identification of internally and externally driven principal dynamic expression patterns of meta-genes (ca-nonical temporal expression trajectories)”.(TIF)

S3 Fig Internally and externally principal dynamic patterns of cross-species conserved gene expression during human breast cancer cell cycle after hormonal stimulation. The horizontal axis represents 12 time points from 0 to 32 hours during a complete mitotic breast cancer cell cycle (E-TABM-631, ArrayExpress). The vertical axis represents the normalized PDP expression with the vector norm equal to one. The internal group is defined as a set of cross-species conserved human genes (i.e., 1132 worm-fly-human orthologs; including 150 orthologous TFs), and the external group consists of 1870 human-specific TFs.
(TIF)

S1 Table Examples of internal and external regulatory networks. (DOCX)
