# Supplementary figures and images for: DREISS: Using State-Space Models to Infer the Dynamics of Gene Expression Driven by External and Internal Regulatory Networks
Source: PLoS Comput Biol. 2016 Oct 19;12(10):e1005146. doi: 10.1371/journal.pcbi.1005146 (PMC5070849; doi:10.1371/journal.pcbi.1005146)

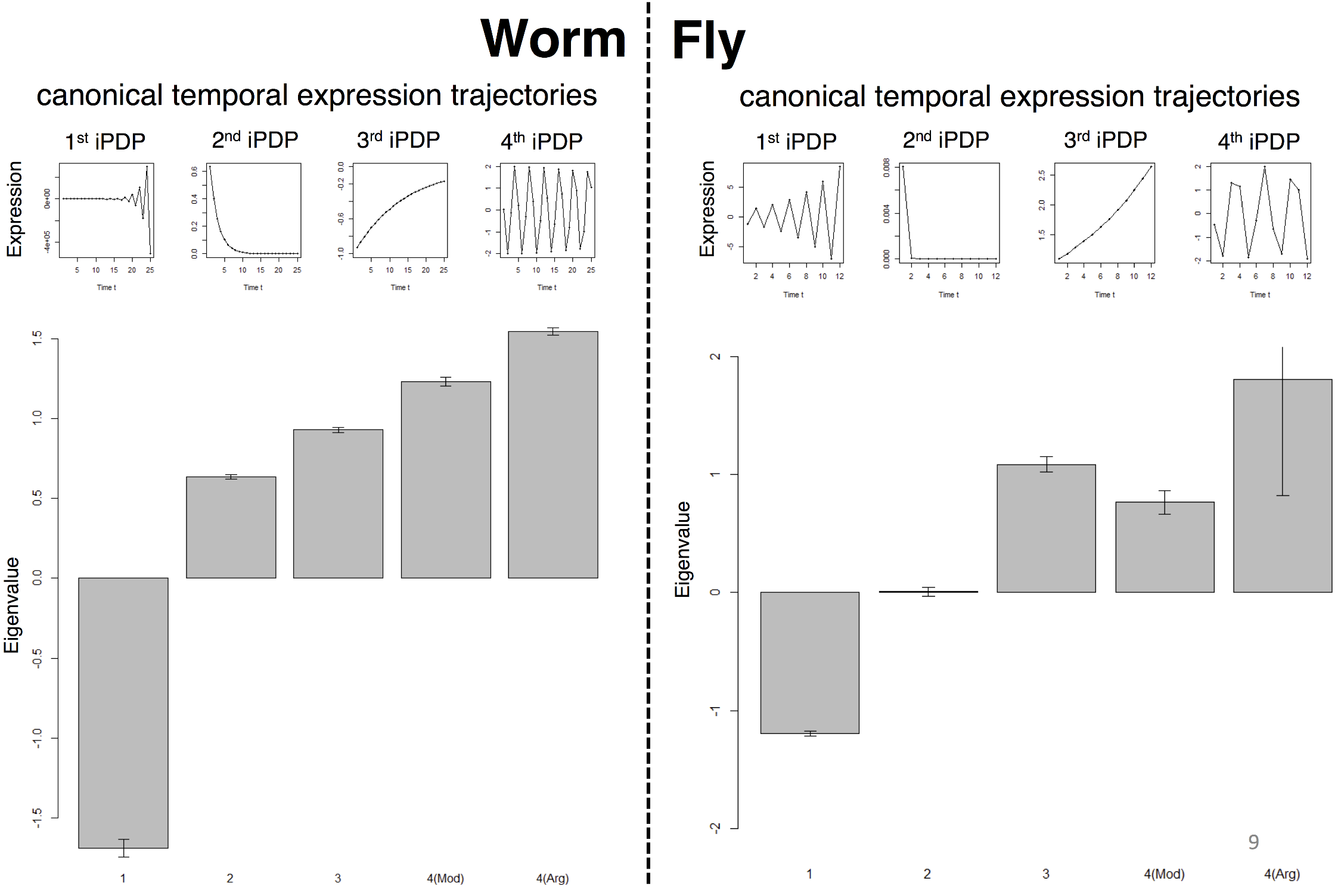

Supplement: S1 Fig — Internal principal dynamic patterns (iPDPs) of orthologs during worm and fly embryonic development. Barplots show the eigenvalues of iPDPs. The error bar for each eigenvalue tells the its variation range. We left one gene out, and calculated eigenvalues for the remaining genes thus obtaining the eigenvalue variations. The curves show the canonical temporal expression trajectories of iPDPs. (TIF) [file pcbi.1005146.s001.tif]

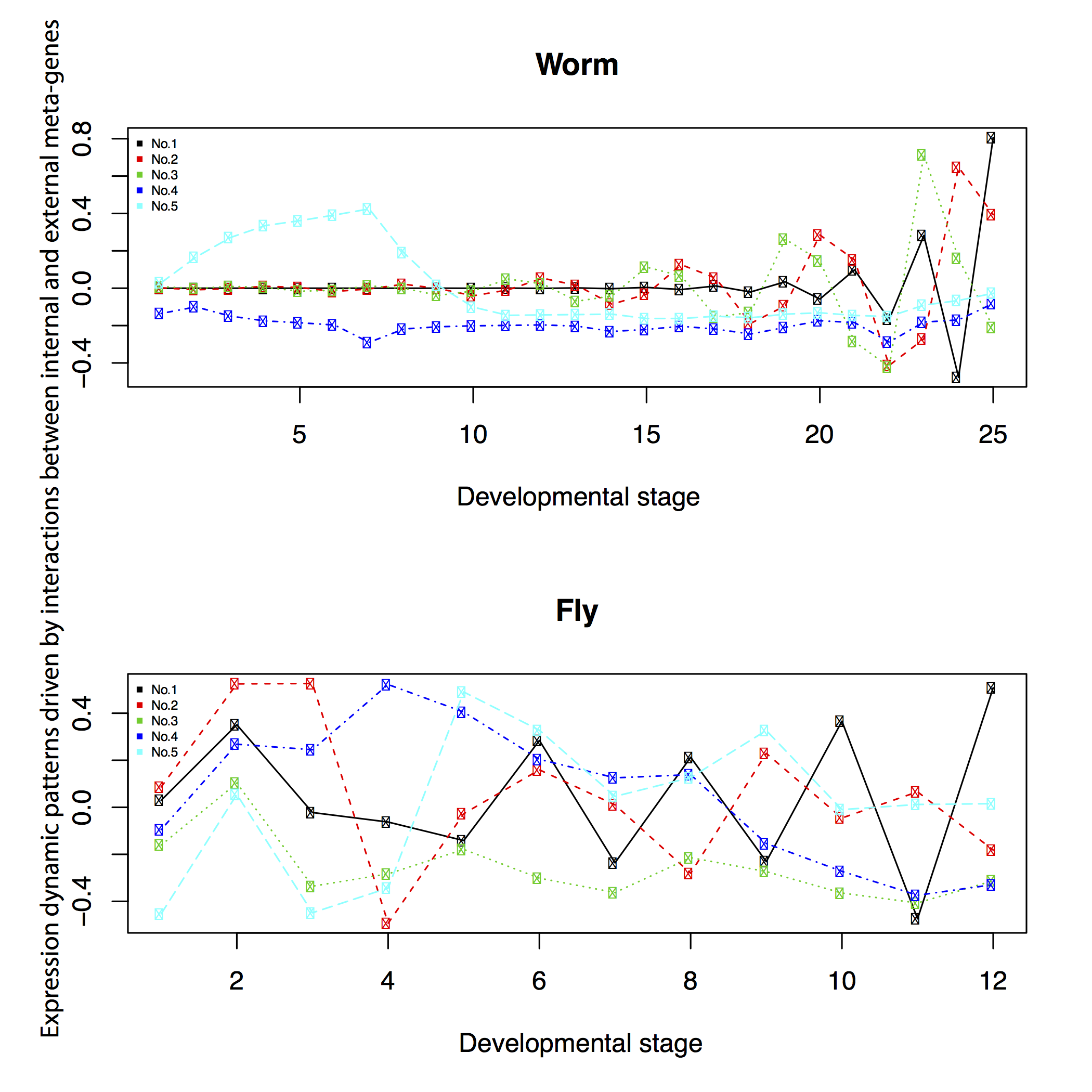

Supplement: S2 Fig — The first five singular vectors (>95% covariance in total) of [X˜tINTER,t=1,2,3,…,T] defined at the end of Section “Identification of internally and externally driven principal dynamic expression patterns of meta-genes (ca-nonical temporal expression trajectories)”. (TIF) [file pcbi.1005146.s002.tif]

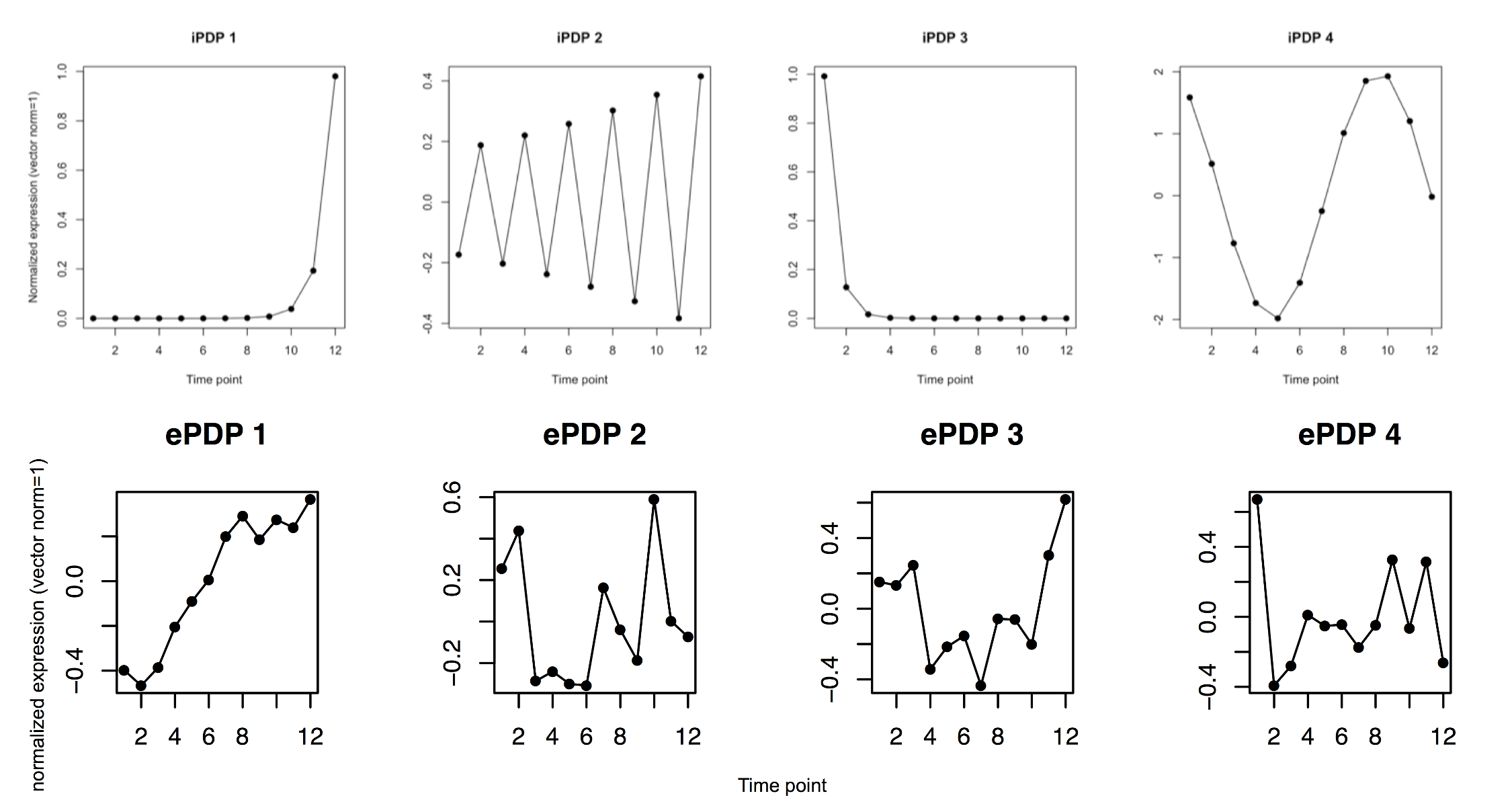

Supplement: S3 Fig — The horizontal axis represents 12 time points from 0 to 32 hours during a complete mitotic breast cancer cell cycle (E-TABM-631, ArrayExpress). The vertical axis represents the normalized PDP expression with the vector norm equal to one. The internal group is defined as a set of cross-species conserved human genes (i.e., 1132 worm-fly-human orthologs; including 150 orthologous TFs), and the external group consists of 1870 human-specific TFs. (TIF) [file pcbi.1005146.s003.tif]
