# Supplementary material for: DREISS: Using State-Space Models to Infer the Dynamics of Gene Expression Driven by External and Internal Regulatory Networks
Source: PLoS Comput Biol. 2016 Oct 19;12(10):e1005146. doi: 10.1371/journal.pcbi.1005146 (PMC5070849; doi:10.1371/journal.pcbi.1005146)
Supplement: S1 Table — (DOCX) [file pcbi.1005146.s004.docx]

Supplemental Table 1 – Examples of internal and external regulatory networks

| Interested system | Internal regulatory network | External regulatory network |
| --- | --- | --- |
| Cross-species conserved genes | Orthologous transcriptional factors (TFs) | Species-specific TFs |
| Protein-coding genes | TFs | micro-RNAs |
| Individual’s protein coding genes | Wild-type TFs | Somatic mutated TFs |
| Protein-coding genes in brain | Commonly expressed TFs | Brain-specific expressed TFs |
| Protein-coding genes in development | House-keeping TFs | Developmental TFs |
